# Proactive coping and social-emotional adjustment among students with and without learning disabilities in Kerala, India

**DOI:** 10.3389/fpsyg.2022.949708

**Published:** 2022-10-06

**Authors:** D. P. Deepthi, Sundaramoorthy Jeyavel, G. Subhasree, Chacko Eapen Jojo

**Affiliations:** ^1^Department of Psychology, School of Social and Behavioural Science, Central University of Karnataka, Kalaburagi, India; ^2^Department of Psychology, School of Social Sciences, Central University of Punjab, Bathinda, India

**Keywords:** learning disabilities, proactive coping, social adjustment, emotional adjustment, educational adjustment, coping skills training

## Abstract

The current study compared the level of proactive coping and social-emotional adjustment of students with and without learning disabilities. In addition to the relationship, influence of proactive coping on social-emotional adjustment of students with and without learning disabilities was also explored. Using a multistage random sampling method researcher selected students with and without learning disabilities in the age group of 15–17 years; each group consists of 150 participants from different high schools in Kerala. The instruments employed in this study were the Proactive Coping Inventory and the Adjustment Inventory for School Students-AISS. Correlation, *t*-test, and regression analysis were used to analyses the data. The students with learning disabilities have found to have lower levels of proactive coping and social emotional adjustment than those without learning disabilities. Further, a positive correlation between social emotional adjustment and proactive coping was also observed. The regression analysis has revealed that proactive coping of students with and without learning disabilities was significantly predicting their adjustment. As students with learning disabilities showing lower proactive coping skills, the study emphasizes the need to enhance proactive coping among students with learning disabilities. Improving proactive coping in both students with and without learning disabilities may help to mitigate social emotional adjustment issues.

## Introduction

Learning disabilities (LD) is an “invisible handicap” which needs more attention in the education system. It is a neurodevelopmental disorder that constitutes a heterogeneous group of disorders related to listening, speaking, reading, writing, reasoning, or mathematical abilities that are thought to be due to the dysfunctionality of the Central Nervous System. LD is not the result of sensory impairment, intellectual disability, serious emotional disturbance, cultural differences, or insufficient or inappropriate instruction, although it may occur along with these conditions (American Psychiatric Association [APA], 1995). Worldwide, 5–15% of children in the school going age group struggle with LD (American Psychiatric Association [APA], 1995). A systematic review of the prevalence studies revealed that 10% of school going children in India and 8% of the general population in Kerala have LD (Suresh and Sebastian, 2003; Singh et al., 2017; Kuriyan and James, 2018).

In addition to academic and cognitive difficulties, many students with LD experience social and emotional difficulties such as high level of peer rejection, loneliness, low sense of coherence, low self-concept, high level depression, and anxiety (Bender and Wall, 1994). Social-emotional development of adolescents is more impaired than it is in the earlier stages. Research shows that adolescents with LD are more likely to be victims of depression and suicide, less satisfied with their peer relations and involved in delinquent activities than their peers without LD (Huntington and Bender, 1993). Some students with LD had reduced self-esteem and perceived competence, which had a negative impact on social behavior and academic achievement (Grolnick and Ryan, 1990). Adolescents with LD who have high levels of anxiety may develop minor somatic complaints and experience high levels of loneliness (Margalit, 1991; Sabornie, 1994). Researchers also suggest that in addition to academic remediation, students with LD need more attention to their social-emotional development training (Kavale and Mostert, 2004; Khodadadi et al., 2017).

Inadequate management of social-emotional problems may increase the risk of later maladjustment (Greenham, 1999). The existing reviews revealed that students with LD experience adjustment issues, particularly social-emotional adjustment (Al-Yagon and Mikulincer, 2004). Some students with LD have been observed to have internalized emotional symptoms of depression and anxiety as well as problematic externalized emotional behaviors like aggression, delinquency, and hyperactivity (Greenham, 1999). They were facing adjustment problems in schools including unsatisfied peer relations, poor relation with teachers and a lack of bonding with the school (Murray and Greenberg, 2001; Charitaki et al., 2018). Research studies conducted in the Indian context supports the adjustment issues of school going students with LD as well (Sharma et al., 2017).

Due to insufficient management of high-level stress, some students with LD develop social-emotional adjustment problems (Hampel and Petermann, 2006; Khutaba, 2014). Reviews have found that students with LD have a higher stress level than their peers without LD since they have more reasons to be stressed and fewer coping skills (Huntington and Bender, 1993; Bender, 2007). Children with LD may have trouble anticipating or forecasting stressful circumstances due to poor metacognitive or executive functioning skills. As a result, the uncertain stress inducing events exacerbate their sensitivity to these stressors (Huntington and Bender, 1993; Bender and Wall, 1994). Students with LD lack inner resources to cope with stressful situations, and low social competence reduces the likelihood of using social resources (Khodadadi et al., 2017). The school environment was stressful for them due to the repeated failure in classrooms, negative attitude of teachers, labeling, peer rejection, and parental pressure (Bryan et al., 2004). If these problems are not recognized and managed earlier, the level of difficulty will increase, and these students may develop stress-related disorders, behavioral problems, suicidal tendencies, depression, and dropping out of school (Kempe et al., 2011; Johnson, 2017).

The presence of LD has an impact on the well-being of parents and primary caregivers of students with LD (Parameswari and Jeryda Gnanajane Eljo, 2017). Recent studies revealed that parents caring for children with developmental disabilities were at risk of mental health issues like high level stress, depressive symptoms, general health issues, and fatigue than parents of children without LD (Sahu et al., 2018; Marquis et al., 2020; Masefield et al., 2020). According to Simon and Easvaradoss (2015) parents of children with LD have a lower quality of life and experience more parenting stress than parents of children without LD. Psychological resources as well as the coping resources of parents had an influence on the social emotional adjustment of students with LD (Al-Yagon and Margalit, 2012). Therefore, the mental health issues of parents also related to the maladjustment of students with LD.

The instructions and interventions which focus on developing coping skills among students with LD can reduce the severity of stress and stress-related problems. Coping can be defined as “constantly changing cognitive and behavioral efforts to manage specific external and/or internal demands that are appraised as taxing or exceeding the resources of the person (Lazarus and Folkman, 1984).” According to reviews, many students with LD have difficulties to use appropriate coping strategies in stressful situations. They use adaptive coping strategies less frequently in a problematic situation because they perceive themselves as less competent, both academically and socially (Shulman et al., 1995; Bagnato, 2017). Instead of using productive coping strategies, they were ignoring the problem as well as unable to appraise the source of the stress which they expected to deal with (Shulman et al., 1995).

Despite the fact that many students with LD struggle with healthy coping, the condition does not impair their ability to choose suitable behaviors to deal with the problem. However, an early age of onset and long-term condition of LD could lead to profound suffering that can compromise the ability to control events, and consequently, favor the adoption of inadequate coping modalities (Bagnato, 2017). However, there are people with LD who adaptively cope with their challenges (Reiff et al., 1995; Raskind et al., 1999). A study on the characteristics of successful students who have LD by Nunez et al. (2005) have found out that a proactive attributional style was associated with positive outcomes. Research in the fields of self-regulation, academic motivation, and attribution has also shown the importance of teaching students who have to LD to be self-aware regarding their condition and proactive in their response to it (Petersen et al., 1993; Alexander et al., 1998). Considering these facts, it is understandable that employing proactive coping strategies would help students with LD in accepting their disability and take on the difficulties as a challenge which alter their mode of function (Firth et al., 2008).

Proactive coping focuses on future-oriented coping. According to Aspinwall and Taylor (1997), proactive coping consists of “efforts undertaken in advance of a potentially stressful event to prevent it or to modify its form before it occurs.” Existing research has focused on reactive coping strategies among students with LD which are concerned with how people cope with the past or ongoing stress (Schwarzer and Taubert, 2002). Proactive coping is different from other forms of coping because it is more focused on future challenges along with tenacious goal pursuit (Schwarzer and Luszczynska, 2008). Anticipatory and preventive coping strategies are not the same as proactive coping. Anticipatory coping deals with the critical event that will occur in the near future. Therefore, it is a short-term engagement with high certainty events, whereas proactive coping entails foreseeing upcoming challenges that are perceived as potentially self-promoting. Preventive coping is the effort to minimize the risk of stressful events in the distant future through building up general resistance resources. Preventive coping focuses on coping to prevent adversity, as opposed to proactive coping, which focuses on coping to promote personal growth. Preventive and proactive coping share some overt behaviors, such as skill development, resource accumulation, physical fitness improvement, and long-term planning. However, proactive coping differs from preventive coping in several ways, including threat assessment, level of worry, and goal management. Proactive coping views threats as challenges, has a lower worry level than preventive coping, and focuses on self-regulatory goal management rather than risk management (Schwarzer and Luszczynska, 2008). Proactive coping uses positive emotional strategies that utilize the resources available to the person and promotes personal growth as well. Since proactive coping is focusing on the future-oriented goal management, positive perception of stress, and formation of opportunities, it is considered as the most beneficial approach than reactive, anticipatory, and preventive coping (Schwarzer and Taubert, 2002).

Research findings reveal that proactive coping is an effective stress management technique (Tharbe, 2006). Individuals are proactive rather than reactive in the sense that they take positive steps and generate possibilities for advancement. The proactive individual strives for the improvement of life and builds up resources that assure progress and quality of functioning. Proactive coping can be considered as an effort to build up resources that facilitate promotion toward challenging goals and personal growth. People may be naturally proactive, or they can learn to be proactive. The benefit of being proactive is nothing but the ability to anticipate future challenges and planning to manage them using all available resources of the individuals (Schwarzer and Luszczynska, 2008; Sohl and Moyer, 2009; Sheikh Hamid et al., 2013).

Proactive coping studies have been conducted in a variety of populations, including adolescents, college students, employees, adults, people with health issues, and the elderly (Gan et al., 2010; Sheikh Hamid et al., 2013; Zambianchi and Ricci Bitti, 2014). Recent research has also studied the effect of proactive coping in the context of the Covid-19 (Chang et al., 2021; Pearman et al., 2021). Successful proactive coping intervention studies conducted on students, adults, and diabetic patients (Bode et al., 2007; Thoolen et al., 2009; Kadhiravan and Kumar, 2012; Kroese et al., 2014; van der Velde et al., 2021).

Many studies have explored proactive coping in adolescents and found that it is an effective stress management technique as well as being associated with adolescent wellbeing, adjustment, and self-efficacy (Tharbe, 2006; Gan et al., 2010; Bogdan et al., 2012; Kadhiravan and Kumar, 2012; Kumar and Bharti, 2018). As the research established a relationship between proactive coping and adjustment among students without LD, understanding the relationship and influence among these two variables in students with LD will benefit the development of interventions and remedial teaching.

There have been relatively few studies on the proactive coping of the LD population. According to Goldberg et al. (2003), proactivity is one of the factors influencing the success of adults with LD. Despite their difficulties, they take charge and employ effective coping strategies. According research finding, many older people use proactive coping more frequently than younger people in a normal population (Sollar and Sollarova, 2009). Hence, successful adults, both with and without LD, may employ proactive coping strategies more than adolescents. Previous research comparing reactive coping styles in adolescents with and without LD revealed that many students with LD use unhealthy coping strategies (Cheshire and Campbell, 1997; Firth et al., 2008; Givon and Court, 2010). Given that proactive coping is a success factor, the main questions being addressed in this study are as follows: (a) To what extent are students with LD proactive? (b) Do they really differ from their peer group on this aspect? (c) How does it relate to the social and emotional adjustment of LD students? Since proactivity is a learnable phenomenon, exploring it will help us to foresee the need of tailoring intervention to help the students with LD to cope with their day-to-day stressors in a better manner.

### Purpose of the present study

The current study focused on assessing the level of proactive coping and social-emotional adjustment of adolescent students with LD, and to compare them with adolescent students without LD. Furthermore, the relationship between proactive coping and social emotional adjustment among students with and without LD are also explored. This study was part of a research to tailor and execute a proactive coping enhancement program targeting adolescent students diagnosed with LD. The findings of this study would be helpful to understand the nature of proactive coping among students with LD and explore the various choices to enhance it to mitigate their social-emotional adjustment. This study examines the prediction of proactive coping on social emotional adjustment. Understanding the differences in proactive coping prediction on social emotional adjustment in students with and without LD helps in the development of effective intervention plans for each group. It is essential for finding ways to foster the best possible growth and adjustment amongst them. There have been studies that show a link between adjustment and proactive coping among students, but less attention has been paid to the relationship with social emotional adjustment. Researchers could not find studies comparing proactive coping in students with and without LD. Since it is the first attempt to look into proactive coping of LD students and their social emotional adjustment, we have proposed a null hypothesis stating that “there is no significant difference in the proactive coping and social-emotional adjustment levels between the scores of adolescent students with and without LD.”

## Materials and methods

### Participants

Participants of this study were selected from three different districts in Kerala, India. The researcher had approached the Education Department of Kerala and gained the information about the total number of students diagnosed with LD in the regular schools. As per the Kerala Education Department report (2019–20), there were 9,679 elementary school students and 10,417 high school and higher secondary school students diagnosed with LD. This study employed multistage random sampling. Among the 14 districts in Kerala Kozhikode, Malappuram, and Trivandrum were randomly selected. The total number of government schools, private aided schools, and private unaided schools in each district were retrieved and 10% of each category of schools in each district were calculated. Around 20 schools from each district were selected randomly, and students with LD were chosen from each school (a limited number of students with LD were identified in each school). Based on the number of LD students in each class, an equal number of students without LD were selected using the fishbowl technique.

This study included 150 students diagnosed with LD and 150 students without LD who were enrolled in regular high schools in Kerala between the ages of 15 and 17. In Particular, students with LD who were diagnosed by certified clinical psychologists in Kerala and all the subtypes of LD have been included in this study. The students with LD as a comorbidity of attention deficit hyperactivity disorder (ADHD) have been excluded.

### Instruments

The current study included students with learning disabilities who are already diagnosed by government clinical psychologists in Kerala. Based on the classroom achievement test teachers select the low scoring students and reported this to the special education teachers in the school. Special education teachers screen these students using a standardized learning disabilities screening tool developed by the Institute of Mental Health and Neurosciences (IMHANS), Kozhikode. Finally, the selected students were assessed and diagnosed by certified clinical psychologists at government hospitals in Kerala.

Proactive coping refers to efforts undertaken in advance of a potentially stressful event to prevent it or to modify its form before it occurs (Aspinwall and Taylor, 1997). To assess the proactive coping among students with and without LD, the researcher used the Proactive Coping Inventory (PCI) developed by Greenglass et al. (2018). PCI was developed to measure different dimensions of the proactive approach to coping and consists of subscales—including proactive coping, reflective coping, avoidance coping, strategic planning, preventive coping, instrumental support seeking, emotional support seeking etc. PCI consists of seven subscales with 55 items. One scale with 14 items measures proactive coping exclusively. The Proactive Coping subscale is a self-report test that assesses goal attainment cognitions and behavior. All items are graded using a 4-Likert-Type scale (not at all true, barely true, somewhat true, and completely true). The score on the proactive coping subscale ranges from 14 to 56. Individuals who score high on the Proactive Coping subscale are perceived to have beliefs that have a strong potential for change, particularly in ways that enhance oneself and one’s environment. PCI subscales have a high internal consistency, as evidenced by reliability measures (α) of 0.85 and 0.80 in the two samples. Furthermore, the scale has high item-total correlations and adequate skewness as a measure of symmetry around the mean. The factorial validity and homogeneity of the model were confirmed using a principal component analysis (Greenglass et al., 2018).

Adjustment Inventory for School Students (AISS) developed by Sinha and Singh (1995) have been used for assessing the social-emotional adjustment of students with and without LD. This 60-item inventory has been designed and developed to determine the student’s social, affective and educational adjustment. This inventory comprises of three subscales—affective, social and educational adjustment, and each subscale consists of 20 items. It’s scored on a 2-point scale of 0 and 1. High score indicates maladjustment and low score indicates the individual’s good adjustment. The developers of this inventory have determined its reliability coefficient through bisection, test-retest and Kuder-Richardson, which were equal to 0.95, 0.93, and 0.94, respectively. The reliability of social, affective, educational and total adjustment equaled 0.92, 0.92, 0.96, and 0.94, respectively.

### Procedure

Using Multistage Random Sampling, the researcher selected the students with and without LD from three districts in Kerala. Three districts were randomly selected out of the 14 districts of Kerala. The total number of government schools, aided schools, and private schools in each district was ascertained. A total of 10% of schools from each category were counted and accordingly schools in each category were randomly selected. The number of students with LD in the selected schools was estimated, and since each school had a small number of LD students, all of them were included in the study. A similar number of students without LD were also selected using fishbowl sampling technique from the same class of each student with LD. The study started after gaining approval from the Education Department in Kerala, India. The district programme officer of Samagra Shiksha Kerala from selected districts provided the researchers with a written authorization letter. Using that authorization letter, researchers gained additional permission from the selected school administration and conducted the study. According to the ICMR’s national ethical guidelines for children, the current study’s assent was waived under the condition that adult informed consent was obtained. Parents of students with and without LD were informed about the study, including its purpose, method, ethical concerns, risks, and benefits. The written consent was obtained from both parents and students prior to data collection. Students’ participation was a personal choice, and they could leave the study at any time. Confidentiality of the participants was assured. Special education teachers assigned time slots for data collection for each student so that their class periods were not disrupted. Furthermore, socio-demographic details were also collected and administered the Proactive Coping Inventory (PCI) and Adjustment Inventory for School Students (AISS). The participants answered the questionnaires in about 45 min. Following that, norms were used to score and interpret the data and appropriate statistical tests were used for the analyses the data. For assuring the confidentiality of the data, only the researchers collected and analyzed the data, and the data files were password protected. Personal information about the participants was not revealed anywhere. Ethical guidelines were strictly followed and participants were treated with dignity. Ethical clearance had been obtained from the affiliated institution of the researchers. The data collection for the study was completed in 4 months, from November 2019 to February 2020.

### Data analysis

The researcher used an independent Sample *t*-test, Karl Pearson correlation test and linear regression to analyze the collected data. The Karl Pearson correlation test was used to analyze the relationship between proactive coping and social-emotional adjustment among students with LD. The same test was also used for students without LD. To understand the contribution of proactive coping to adjustment of students with and without LD, linear regression analysis was used. An independent sample *t*-test was used for comparing the proactive coping and social-emotional adjustment between students with and without LD the group difference was also found out using the independent sample *t*-test.

## Results

The demographic details of the participants in the study showed that the mean age of students with LD was 15.79 and students without LD were 15.59. Among 150 students with LD, 129 are boys (86%), and 14 are girls (14%). Among students without LD, 72 are boys (48%) and 78 are girls (52%). Participants are belongs to the class 9th, 10th, 11th, and 12th in the various government (54%) and private aided (46%) schools in Kerala.

### Relationship between proactive coping and social-emotional adjustment

Pearson’s correlation analysis was separately conducted to examine the relationship between proactive coping and social-emotional adjustment among students with and without LD (Table 1). The result shows a significant statistical correlation between proactive coping and social-emotional adjustment of students with LD. There was a negative correlation between proactive coping and the three variables in adjustment, which were as follows: social adjustment (*r* = –0.382, *p* < 0.01), emotional adjustment (*r* = –0.209, *p* < 0.05), and educational adjustment (*r* = –0.217, *p* < 0.01). A similar pattern of results were received from the analysis of students without LD. There was a negative correlation between proactive coping and social-emotional adjustment of students without LD. The Pearson correlation coefficient between proactive coping and the different forms of adjustment is as follows, social adjustment (*r* = –0.287, *p* < 0.01), emotional adjustment (*r* = –0.283, *p* < 0.01) and educational adjustment (*r* = –0.292, *p* < 0.01). The findings showed a negative relationship between proactive coping and the three adjustment factor indicating that as an individual’s proactive coping score increases, their maladjustment score decreases. The person who has high proactive coping skills may be less maladjusted.

**TABLE 1:** Correlational analysis: Proactive coping and adjustment among students with and without learning disabilities.

Variables	Proactive coping			
	Students with LD	*p*	Students without LD	*p*
Social adjustment	–0.382	0.001	–0.287	0.001
Emotional adjustment	–0.209	0.010	–0.283	0.001
Educational adjustment	–0.217	0.008	–0.292	0.001

### Contribution of proactive coping to adjustment of students with learning disabilities

In examining the contribution of proactive coping on social-emotional adjustment, a series of linear regression analysis was conducted for each of the adjustments (social, emotional, and educational) separately for students with and without LD (Table 2). The regression analysis revealed that proactive coping of students with LD significantly predicted their social adjustment 14.6%, *F*(1, 148) = 25.277, *p* < 0.01, emotional adjustment 4.4%, *F*(1, 148) = 6.738, *p* < 0.05 and educational adjustment 4.7% *F*(1, 148) = 7.285, *p* < 0.01.

**TABLE 2:** Linear regression analysis of proactive coping and adjustment among students with and without learning disabilities.

Independent variable	Dependent variable	Students with LD			Students without LD		
		Standardized β	*t*-value	Model summary	Standardized β	*t*-value	Model summary
Proactive coping	Social adjustment	–0.382	5.028**	*R*^2^ = 0.146 *F* = 25.277 Sig = 0.001	–0.287	3.644**	*R*^2^ = 0.082 *F* = 13.276 Sig = 0.001
	Emotional adjustment	–0.209	2.596*	*R*^2^ = 0.044 *F* = 6.738 Sig = 0.010	–0.283	3.594**	*R*^2^ = 0.080 *F* = 12.916 Sig = 0.001
	Educational adjustment	–0.217	2.699**	*R*^2^ = 0.047 *F* = 7.285 Sig = 0.008	–0.292	3.720**	*R*^2^ = 0.085 *F* = 13.835 Sig = 0.001

**p < 0.01, *p < 0.05.

Among the three domains of adjustment in students with LD proactive coping has more influence on social adjustment than emotional and educational adjustment. Proactive coping explains approximately 14% variance in social adjustment and 4% variance in both emotional and educational adjustment. Comparatively standardized beta value is also high in social adjustment which shows the strength of the effect of proactive coping on social adjustment which is higher than emotional and educational adjustment.

In the same way, regression analysis of students without LD also showed that proactive coping significantly predicted their social adjustment 8.2%, *F*(1, 148) = 13.276 *p* < 0.01, emotional adjustment 8%, *F*(1, 148) = 12.916, *p* < 0.01 and educational adjustment 8.5%, *F*(1, 148) = 13.835, *p* < 0.01. Maximum 8% of the variance in the adjustment that the proactive coping explains collectively. Among students without LD *R*^2^ and standardized beta values are almost the same for social emotional and educational adjustment. The contribution of proactive coping on social adjustment is greater in students with LD than in students without LD. However, the contribution of proactive coping on emotional and educational adjustment is lower in students with LD compared to those without LD.

### Proactive coping and social emotional adjustment: Group difference

A *t*-test analysis was performed to determine the difference in proactive coping between students with and without LD (Table 3). The results reveal that there is a significant difference in proactive coping between students with and without LD (*t* = 14.703, *p* < 0.01). Compared to students without LD (*m* = 42.76), the use of proactive coping was much lesser in students with LD (*m* = 33.16).

**TABLE 3:** ‘t’ test comparison of proactive coping and social–emotional adjustment between students with and without learning disabilities.

Variables	Students with LD		Students without LD		*t*-value
	Mean	*SD*	Mean	*SD*	
Proactive coping	33.16	6.798	42.76	4.210	14.703**
Social adjustment	6.47	2.319	4.98	1.954	6.004**
Emotional adjustment	6.44	2.387	4.82	2.821	5.369**
Educational adjustment	9.01	3.128	7.66	3.381	3.598**

**p < 0.01.

The comparison of social-emotional adjustment of students with and without LD was also examined using the *t*-test analysis (Table 3). The results revealed a significant difference emerging in the student groups on the following three adjustments—social adjustment (*t* = 6.004, *p* < 0.01), emotional adjustment (*t* = 5.369, *p* < 0.01), and educational adjustment (*t* = 3.598, *p* < 0.01). From the table, we can learn that the students with LD are more maladjusted than students without LD.

## Discussion

The first objective of this study was to find the relationship between proactive coping and social-emotional adjustment among students with and without LD. The result of this study revealed a significant negative correlation between proactive coping and social-emotional maladjustment, in both students with and without LD, which means that when the proactive coping increases, the maladjustment decreases. This finding suggests that increased proactive coping strategies may help to reduce social-emotional adjustment problems in students with and without LD. The results of this study are consistent with findings in the literature demonstrating a link between coping and adjustment. Gan et al. (2010) revealed the positive relationship between proactive coping and adjustment of college students; how it influences the adjustment of college students during their university life in such a way that students who possessed more proactive coping strategies adjusted better. The transactional theory of coping assumes that successful coping involves an ability to adjust and change strategies in a way that facilitates positive outcomes (Lazarus and Folkman, 1984). Few research has been undertaken to examine the relationship between coping and adjustment among students with LD. The effect of coping intervention on the social-emotional adjustment of students with LD was explored in the experimental study of Khodadadi et al. (2017) and they discovered that teaching coping strategies improved the social-emotional adjustment and educational adjustment of students with LD.

The current study found the contribution of proactive coping to social-emotional adjustment through a series of linear regression analyses. The result revealed that proactive coping by students with LD significantly predicted the social, emotional, and educational adjustments. The change in proactive coping could influence a change in social-emotional adjustment of students with LD. Proactive coping has maximum 14% of statistical influence on the variations in social, adjustment and the remaining variance may be contributed by other factors not in the focus of current study. The proactive coping of students without LD also significantly predicted 8% of the social adjustment. While comparing the standardized “β” we could the see higher proportion of influence found in students with LD which signifies the better role of proactive coping in determining social adjustment of LD students. Overall, encouraging students to use proactive coping strategies is likely to help to mitigate the social, emotional maladjustment.

The second objective of this study was to compare the proactive coping and social-emotional adjustment between students with and without LD. The result of the *t*-test shows that there is a significant difference between proactive coping among students with and without LD. Compared to the students without LD, the level of proactive coping was significantly less among students with LD. Previous research has found that many students with LD have poor coping skills based on peer comparison, demonstrating that instead of healthy coping, many of them used unhealthy and unproductive coping styles in stressful situations than their peers without LD (Cheshire and Campbell, 1997; Givon and Court, 2010). Bagnato (2017) found that students with LD were using adaptive coping less frequently than students without LD. Instead of active and distraction coping, some students with LD adopt aggressive behavior and escaping coping more frequently than their peers without LD. Similarly, Firth et al. (2010) reported that many adolescents with LD tended to use more non-productive coping strategies such as ignoring difficulties, not coping and self-blame than effective coping strategies like working hard and focusing on positives when compared to peer without LD.

The reviews present a variety of perspectives on the causes of poor coping skills in students with LD. Studies found that, deficiencies in cognitive and social skills were one of the causes for lesser use of adaptive coping among adolescents with LD. These findings may be due to, cognitive and social skills impairments obstruct their ability to assess the problem accurately and engage in information seeking behavior, respectively (Shulman et al., 1995; Geisthardt and Munsch, 1996; Bender et al., 1999; Bagnato, 2017). Similar research indicates that cognitive deficits in the area of attention, memory, perceptual skills, and motor skills have a negative impact on the adaptive coping of students with LD (Torgesen and Houck, 1980). According to Geisthardt and Munsch (1996), in stressful situations, students with LD use cognitive avoidance as a coping mechanism more frequently than students without LD. The delayed cognitive development may have an impact on the use of coping skills that require complex cognitive manipulations. As a result, compared to their peer group, students with LD use coping mechanisms such as cognitive avoidance and emotional discharge which require simple cognitive processes. When they were provided with the simple alternatives to manage their stressful situation, they may able to practice it with their limited cognitive resources. Likewise, the lack of control over the stressful situation leads to the use of denial as a coping mechanism among students with LD. Enhancing the control over their choice and action may also enable them to mitigate the non-adaptive coping mechanisms.

These are not the experiences of all students with LD; many students with LD have overcome obstacles and achieved success in their lives. Nunez et al. (2005) have reported that successful students with LD have adaptive attributional profile rather than helpless attributional profile. Successful students with LD had more positive self-concept, more confidence in their abilities, and they will take more responsibility for their performance and demonstrate higher levels of engagement, effort, and persistence. According to Goldberg et al. (2003) longitudinal study, the success attributes in students with LD include self-awareness, proactivity, perseverance, appropriate goal setting, effective use of social support systems, and emotional stability. Furthermore, successful LD students believed they had the ability to shape their own future and influence the course of their lives. As proactive coping is a future oriented coping strategy student with LD can anticipate their potential stress and cope with it using their personal resources. Hence, the use of proactive coping strategies would be beneficial to the success of students with LD. Unfortunately, the finding of the current study showed that students with LD using lower level of proactive coping strategy than their peers without LD. Examining the reason for this based on the reviews, it is assumed that many students with LD may have difficulty performing some stages of proactive coping. Resource accumulation, identification of potential stressors, initial appraisal, initial coping efforts, elicitation, and feedback use are the five stages of proactive coping. Reviews showed that many adolescents with LD were less able to appraise their source of stress and ask for help (Shulman et al., 1995; Bagnato, 2017). They were demonstrated a lower level of resilience, indicating inadequate coping skills and a lack of inner resources to deal with these stressful circumstances (Panicker and Chelliah, 2016). Furthermore, they have a negative self-image and consider themselves as less competent as a result of repeated academic failure and negative attitudes from peers and school members. Hence, they are lacking the skills of approaching the problem, identifying it and taking steps to solve the problems. Their pessimistic attitude toward success leads to a withdrawal from stressful situations (Singer, 2005). These conditions may make it difficult for students with LD to use proactive coping strategies.

The result of the *t*-test revealed that the students with LD showed significantly higher social-emotional maladjustment as compared to their peers without LD. Existing reviews support the findings that students with LD experience extra adjustment issues, especially social-emotional adjustments, than their peers without LD (Al-Yagon and Mikulincer, 2004; Tamannaeifar and Nezhad, 2014). This is because they have academic and social difficulties, high levels of internalizing emotional symptoms of depression, anxiety, and problematic externalized emotional behavior like aggression, delinquency etc. (Greenham, 1999). There are several important factors which are necessary for the social-emotional development and contribute to the individual adjustment of the student, but frequent failures in academic performances, negative attitude of peers, teachers, and parents, lack of social competence, a deficit in social skills lead to a host of social and emotional problems among students with LD (Hoglund and Leadbeater, 2004).

Compared to other peer groups, many students with LD showed social adjustment problems like lack of social skills, poor self-confidence, bad performance in school, poor relationship with peers and teachers, a lack of bonding with the school (Murray and Greenberg, 2001; Charitaki et al., 2018). Adolescents with LD face more adjustment problems in the period of transition in schools than other peer groups because they already have emotional, social, and psychological difficulties (Accariya and Khalil, 2016).

The social-emotional adjustment of students with LD was influenced by family factors like their attachment to family, parenting, coping and emotional resources of parents. The positive family environment reduces the maladjustment problems and helps them to adjust with their surrounding (Al-Yagon and Mikulincer, 2004; Barkauskiene, 2009; Al-Yagon, 2011). Studies conducted in the Indian context also revealed that compared to other peer groups, students with LD showed more social-emotional adjustment problems (Sharma et al., 2017). Also, they exhibited a low level of self-esteem and self-concept than other peer groups (Parshurami, 2015; Pandey, 2017).

The current study found that students with LD have lower levels of proactive coping and social emotional adjustment than their peers without LD. These students may require more attention in school in order to overcome these issues. In addition to academic remediation, school counselors can provide interventions to reduce social and emotional problems and improve proactive coping. Enhancing social skills and problem-solving abilities among these students would reduce peer rejection and bullying. Furthermore, positive student-teacher relationship improves academic performance and fosters positive behavioral skills (Alzahrani et al., 2019). It is also registered that proactive coping could predict outcome such as functional independence, life satisfaction and engagement (Sohl and Moyer, 2009).

As cognitive deficits exist in the person with LD, the same kind of stressors will again manifest in the future and affect their adult life as well. Hence, it is preferable to learn healthy coping mechanisms in order to overcome these issues. Existing reviews focused on reactive coping among students with LD, but current study suggest that proactive coping would be beneficial for them because, rather than a negative appraisal, they can perceive their disability as a challenge and use their available resources for personal growth. Even though it is the first study to explore proactive coping of adolescents with LD and without LD, I has added our understanding of the deficit in proactivity among adolescents with LD. By establishing goals, these students will be able to deal with problems in their lives rather than simply adapting to the situation. Although the research design will not allow causal conclusions to be drawn, future research could explore whether proactive coping can improve social emotional adjustment among students with LD. Many students with LD may benefit from proper adjustment and stress coping in order to minimize school dropouts and other psychological disorders. Therefore, organizing school-based initiatives to orient them toward facing distress through proactive coping would benefit their mental health and well-being at large.

## Conclusion

Proactive coping is an effective stress management technique that creates opportunities for personal growth. Hence, students could use proactive coping to expect future challenges and can plan to manage them using all available resources. Compared to the other peer groups, many students with LD face more stressful situations in schools. The inadequate management of their stress may lead to different negative outcomes. So, the students with LD could be given opportunities to use their proactive coping strategies which can help them to alter their mode of function, to accept their disability and take on the difficulties as a challenge. The current study revealed that compared to other peer groups, students with LD use low levels of proactive coping in a stressful situation, as well as poor social-emotional adjustment. Since the proactive coping is low among students with LD, incorporating an intervention to increase proactive coping in remedial education and special education curriculum may assist LD students in overcoming stress-related challenges and achieving success.

## Limitation and recommendations

The current study had used PCI with the 14 items subscale to assess the proactive coping. Further research could include the other subscales such as reflective coping, avoidance coping, strategic planning, preventive coping, instrumental support seeking, and emotional support seeking, to understand the dominant coping styles of students with and without LD. Another limitation of this study is the research design, i.e., non-experimental design where we could not draw any causal conclusion.

Future research should look into ways to improve proactive coping among students with LD, as well as the effect of proactive coping interventions on the social emotional adjustment of students with LD.

## Data availability statement

The raw data supporting the conclusions of this article will be made available by the authors, without undue reservation.

## Ethics statement

This study was approved by the Departmental Research Ethics Board, Department of Psychology, School of Social and Behavioral Sciences, Central University of Karnataka, India (Ethical clearance number: CUK/DREB/Psy/EC-07/2018-191, dated:17-11-2019). Written informed consent to participate in this study was provided by the participants’ legal guardian/next of kin.

## Author contributions

All authors contributed equally to the conception and execution of the research work and preparation of the manuscript.
